# Supplementary material for: Mechanisms governing the pioneering and redistribution capabilities of the non-classical pioneer PU.1
Source: Nat Commun. 2020 Jan 21;11:402. doi: 10.1038/s41467-019-13960-2 (PMC6972792; doi:10.1038/s41467-019-13960-2)
Supplement: Supplementary file 7 — Source data [file 41467_2019_13960_MOESM7_ESM.zip › Source_Data/FigureS2/FigureS2B_MotifScanOutput/KmeansCluster9/homerResults/motif10.similar.html]

motif10

## Information for motif10

G
C
A
T
G
C
A
T
C
G
T
A
T
G
C
A
A
T
G
C
T
A
G
C
C
T
G
A
G
T
A
C
G
C
T
A
  
Reverse Opposite:  

C
G
A
T
C
A
T
G
G
A
C
T
A
T
C
G
T
A
C
G
A
C
G
T
G
C
A
T
C
G
T
A
C
G
T
A
  

|  |  |
| --- | --- |
| p-value: | 1e-40 |
| log p-value: | -9.396e+01 |
| Information Content per bp: | 1.580 |
| Number of Target Sequences with motif | 298.0 |
| Percentage of Target Sequences with motif | 13.09% |
| Number of Background Sequences with motif | 2482.6 |
| Percentage of Background Sequences with motif | 5.38% |
| Average Position of motif in Targets | 97.8 +/- 55.5bp |
| Average Position of motif in Background | 99.9 +/- 63.4bp |
| Strand Bias (log2 ratio + to - strand density) | -0.1 |
| Multiplicity (# of sites on avg that occur together) | 1.09 |
| Motif File: | file (matrix) reverse opposite |

### Similar de novo motifs found

|  |  |  |  |  |  |  |  |
| --- | --- | --- | --- | --- | --- | --- | --- |
| Rank | Match Score | Redundant Motif | P-value | log P-value | % of Targets | % of Background | Motif file |
| 1 | 0.943 | G C A T A C T G A C G T T C A G A C T G A C G T G A C T G C T A | 1e-35 | -80.913443 | 14.76% | 6.96% | motif file (matrix) |
| 2 | 0.932 | C T A G A C G T C T A G C A T G A C G T G C A T C G T A | 1e-33 | -76.217870 | 11.03% | 4.61% | motif file (matrix) |
| 3 | 0.887 | G C A T C G T A C G T A T G A C G A T C T G C A T G A C G C T A T C G A T G A C | 1e-23 | -53.416301 | 5.05% | 1.61% | motif file (matrix) |
| 4 | 0.826 | G T C A C G A T T A C G G A C T T C A G T A C G A C G T A G C T C G T A C T A G | 1e-20 | -48.228471 | 17.93% | 11.00% | motif file (matrix) |
| 5 | 0.802 | A C G T A C T G A C G T A C T G T A C G A C G T A C G T A G C T A G C T A T C G G C T A A C G T | 1e-14 | -34.161452 | 2.86% | 0.84% | motif file (matrix) |
| 6 | 0.846 | G C A T G A C T G A C T C G A T C G T A G C T A A T G C G T A C T G C A G A T C G C T A | 1e-14 | -32.337859 | 3.82% | 1.43% | motif file (matrix) |
